# Involvement of neutrophils in machineries underlying the rupture of intracranial aneurysms in rats

**DOI:** 10.1038/s41598-020-74594-9

**Published:** 2020-11-17

**Authors:** Mika Kushamae, Haruka Miyata, Manabu Shirai, Kampei Shimizu, Mieko Oka, Hirokazu Koseki, Yu Abekura, Isao Ono, Kazuhiko Nozaki, Tohru Mizutani, Tomohiro Aoki

**Affiliations:** 1 Department of Molecular Pharmacology, Research Institute, National Cerebral and Cardiovascular Center, 6-1 Kishibeshinmachi, Suita City, Osaka 564-8565 Japan; 2 Core Research for Evolutional Science and Technology (CREST) from Japan Agency for Medical Research and Development (AMED), National Cerebral and Cardiovascular Center, 6-1 Kishibeshinmachi, Suita City, Osaka Japan; 3 Department of Neurosurgery, Showa University School of Medicine, 1-5-8 Hatanodai, Shinagawa-ku, Tokyo, Japan; 4 Department of Neurosurgery, Shiga University of Medical Science, Seta Tsukinowa-Cho, Otsu City, Shiga Japan; 5 Omics Research Center, National Cerebral and Cardiovascular Center, 6-1 Kishibeshinmachi, Suita City, Osaka Japan; 6 Department of Neurosurgery, Kyoto University Graduate School of Medicine, 54 Kawahara-cho Shogoin, Sakyo-ku, Kyoto, Japan; 7 Department of Neurosurgery, Tokyo Women’s Medical University, 8-1 Kawata-cho, Shinjyuku-ku, Tokyo, Japan; 8 Department of Neurosurgery, The Jikei University School of Medicine, 3-25-8 Nishishimbashi, Minato-ku, Tokyo, Japan

**Keywords:** Cell biology, Molecular biology, Diseases, Medical research

## Abstract

Subarachnoid hemorrhage due to rupture of an intracranial aneurysm has a quite poor prognosis after the onset of symptoms, despite the modern technical advances. Thus, the mechanisms underlying the rupture of lesions should be clarified. To this end, we obtained gene expression profile data and identified the neutrophil-related enriched terms in rupture-prone lesions using Gene Ontology analysis. Next, to validate the role of neutrophils in the rupture of lesions, granulocyte-colony stimulating factor (G-CSF) was administered to a rat model, in which more than half of induced lesions spontaneously ruptured, leading to subarachnoid hemorrhage. As a result, G-CSF treatment not only increased the number of infiltrating neutrophils, but also significantly facilitated the rupture of lesions. To clarify the mechanisms of how neutrophils facilitate this rupture, we used HL-60 cell line and found an enhanced collagenolytic activity, corresponding to matrix metalloproteinase 9 (MMP9), upon inflammatory stimuli. The immunohistochemical analyses revealed the accumulation of neutrophils around the site of rupture and the production of MMP9 from these cells in situ. Consistently, the collagenolytic activity of MMP9 could be detected in the lysate of ruptured lesions. These results suggest the crucial role of neutrophils to the rupture of intracranial aneurysms; implying neutrophils as a therapeutic or diagnostic target candidate.

## Introduction

The rupture of intracranial aneurysms (IAs) accounts for 85% of spontaneous subarachnoid hemorrhage (SAH)^1^. The incidence of SAH is 9.1/100,000 people per year in a population-based study, but there is a remarkable regional variation^1^. It is well-recognized that SAH incidence is higher in the Finnish (19.7/100,000 people per year) and the Japanese (22.7/100,000 people per year)^1^. SAH has a considerable negative impact on a society due to its high mortality or morbidity. SAH occasionally causes sudden death, even in young people, e.g. 8.3% of affected people die at the site of the onset, without being subjected to medical care^2^. Even if SAH patients receive a proper treatment, 29.7% of them experiences severe morbidity (modified Rankin Scale > 2) and 12.1% die^2^.

Considering the above devastating outcome of SAH after the onset, the development of a novel therapeutic strategy based on the correct understanding of the machinery underlying the rupture of IAs is mandatory for public health. A rat model of IA, in which induced lesions spontaneously and frequently rupture, resulting in SAH, has recently been reported^3,4^. As histopathological examinations of this model have revealed a close similarity with human IAs (e.g. remarkable degenerative changes in media, infiltration of inflammatory cells, or loss of endothelial cells^5–7^), this rat model has, presumably, a similar machinery as that of humans for the regulation of the rupture of lesions. Thus, in this study, we examined the differences in the comprehensive gene expression profile data between rupture-prone IA lesions and the remaining arterial walls in the circle of Willis, to explore differentially represented cascades that are mediating candidates for the rupture of lesions.

## Materials and methods

### IA models of rats and histological analysis of induced IA

All of the following experiments, including animal care and use, complied with the National Institute of Health’s Guide for the Care and Use of Laboratory Animals, and were approved by the Institutional Animal Care and Use Committee of National Cerebral and Cardiovascular Center. The present manuscript adheres to the ARRIVE (Animal Research: Reporting of In Vivo Experiments) guidelines for reporting animal experiments.

Ten-week-old female Sprague − Dawley (SD) rats were purchased from Japan SLC (*Slc:SD*, n = 69, Shizuoka, Japan). Animals were maintained on a light/dark cycle of 12 h/12 h and had free access to chow and water. To induce IAs, rats were subjected to a bilateral ovariectomy, a ligation of the left carotid artery, the right external carotid artery, and the right pterygopalatine artery, and systemic hypertension was achieved by the combination of a high salt diet and the ligation of the left renal artery^4^ under general anesthesia, which was induced by the combination of an intraperitoneal injection of pentobarbital sodium (50 mg/kg) with inhalation of isoflurane (1.5 ~ 2%). Immediately after surgical manipulations, animals were fed chow containing 8% sodium chloride and 0.12% 3-aminopropionitrile (Tokyo Chemical Industry, Tokyo, Japan), an irreversible inhibitor of Lysyl Oxidase catalyzing the cross-linking of collagen and elastin. The animals that died within one week after surgical manipulations were excluded from the analyses. The animals were deeply anesthetized, using an intraperitoneal injection of pentobarbital sodium (200 mg/kg), and transcardially perfused with 4% paraformaldehyde solution, at 56th or 63rd post-surgery for RNA sequencing analysis or histopathological examination, respectively. The circle of Willis was then stripped from the brain surface and an IA lesion induced at an anterior communicating artery or a posterior communicating artery was dissected as a rupture-prone lesion, in case SAH was not macroscopically detected. All the animals that died during the observation period were autopsied to examine the onset of SAH. If SAH was observed, the ruptured IA lesions were stripped and histopathologically examined. Histopathological examination was performed after Elastica van Gieson staining or Azan staining.

### G-CSF treatment

G-CSF (300 μg/kg, MOCHIDA PHARMACEUTICAL CO., LTD., Tokyo, Japan) was subcutaneously administered to rats twice a week, from the 28th day to the 56th day after surgical manipulation. The specimens of IA lesions were then harvested at the 63rd day. The number of myeloperoxidase (MPO)-positive cells or CD68-positive cells was calculated as the cell count present within the area of 1 mm^2^ around the dome of the induced IAs. The dose of G-CSF administered to rats was determined based on the preliminary dose–response analyses.

### RNA purification and RNA sequencing analysis

Dissected rupture-prone aneurysms and the remaining circle of Willis from the same animal were grinded and homogenized in liquid nitrogen at the 63rd day after surgical manipulations. The total RNA was isolated from homogenized samples using an RNeasy fibrous tissue mini kit (QIAGEN, Hilden, Germany) with an on-column DNase treatment, according to the manufacturer’s instructions. The quantity of each total RNA sample was measured using a NanoDrop (Thermofisher, Waltham, MA), and its quality was assessed by using the RNA integrity number (RIN) on an Agilent 4200 TapeStation (Agilent, Santa Clara, CA). The libraries obtained from the purified RNA samples (500 ng) were then prepared using a TruSeq stranded mRNA sample preparation kit (Illumina, San Diego, CA), for the RNA sequencing analyses. Paired-end sequencing (2 × 75 base pair) was performed on a NextSeq500 (Illumina). Each read was then mapped to the *Rattus norvegicus* reference genome (Rnor6) using CLC genomics workbench (version 11, QIAGEN). Differential expression analyses, including principal component analysis and gene ontology (GO) analysis, were performed using the RNA-Seq tool, one similar to the DESeq and the edgeR package, in CLC genomics workbench. The genes whose expression reached a fold change over 1.5 in aneurysm lesions, compared with that in the remaining circle of Willis, were considered to be over-expressed, respectively.

All the raw data from RNA sequencing analysis was deposited to Gene Expression Omnibus (https://www.ncbi.nlm.nih.gov/geo/) (GEO accession: GSE161044).

### Immunohistochemistry

At the indicated time after the aneurysm induction, 5-µm-thick frozen sections were prepared. In some experiments, sections that were prepared in the previous study were also used^4^. After blocking with 3% donkey serum (Jackson ImmunoResearch, Baltimore, MD), the slices were incubated with primary antibodies, followed by incubation with the secondary antibodies conjugated with a fluorescence dye (Jackson ImmunoResearch). Finally, fluorescent images were acquired using a confocal fluorescence microscope system (FV1000 or FV3000, Olympus, Tokyo, Japan).

The following primary antibodies were used: Cy3-conjugated mouse monoclonal anti-α-smooth muscle actin (SMA) antibody (#C6198, Sigma-Aldrich, St. Louis, MO), mouse monoclonal anti-rat CD68 antibody (#ab31630, Abcam, Cambridge, UK), rabbit polyclonal anti-MMP9 antibody (#ab38898, Abcam), rabbit polyclonal anti-myeloperoxidase (MPO) antibody (#ab9535, Abcam), rabbit polyclonal anti-GRO alpha (CXCL-1) antibody (#ab86436, Abcam), and rabbit polyclonal anti-Histone H3 (citrulline R2 + R8 + R17) antibody (#ab5103, Abcam).

### Cell line

HL-60 cell line was used as a model of neutrophils, was purchased from ATCC (#CCL-240, Manassas, VA), and maintained in Dulbecco's Modified Eagle's Medium (DMEM) supplemented with 20% fetal bovine serum (Sigma-Aldrich).

### Quantitative real time (RT)-PCR analysis of cultured cells

HL-60 cells were stimulated with recombinant TNF-α (100 ng/mL, R&D SYSTEMS, Minneapolis, MN) for 90 min. The total RNA was then purified from stimulated cells and reverse-transcribed by using an RNeasy Mini Kit (QIAGEN) and a High-capacity cDNA Reverse Transcription Kit (Life Technologies Corporation, Carlsbad, CA), according to the manufacturers’ instructions. For quantification of gene expression, RT-PCR was performed on a Real Time System CFX96 (Bio-rad, Hercules, CA) using a SYBR Premix Ex Taq II (TAKARA BIO INC., Shiga, Japan). *ACTB* expression was used as the internal control. For quantitation, the second derivative maximum method was used for determining the crossing point.

The primer sets used are listed in Supplementary Table S1.

### Gelatin Zymography

HL-60 cells were stimulated using recombinant TNF-α (100 ng/mL) for 90 min. The supernatant was then prepared and the collagenolytic activity of the supernatant was examined using gelatin zymography, according to the manufacturer’s instructions (Gelatin Zymography Kit, Cosmo Bio Co., LTD., Tokyo, Japan). In a rat model, ruptured IA lesions were harvested and grinded in liquid nitrogen. Specimens were then lysed and subjected to gelatin zymography, as per manufacturer’s instructions (Gelatin Zymography Kit, Cosmo Bio Co., LTD.).

### The concentration of TNF-α or PGE_2_ in the culture supernatant

HL-60 cells were stimulated with recombinant TNF-α (100 ng/mL) or LPS (10 μg/ mL, SIGMA, Lot # 123M4052V) for 5 h. In some experiments, cells were pre-treated with indomethacin for 15 min (100 nM, Wako). The supernatant was then prepared and the concentration of TNF-α or PGE_2_ in the supernatant was examined, as per manufacturer’s instructions (Quantikine ELISA for human TNF-α, R&D SYSTEMS, or Prostaglandin E_2_ EIA Kit, Cayman Chemical, Ann Arbor, MI).

### Statistical analysis

All the data obtained was used for the analyses. Data are shown as box-and-whisker plots, which represent the maximum value, the minimum value, the median value (vertical line), and the interquartile range of each data set. Statistical comparisons between the 2 groups were conducted using a Mann − Whitney U test and comparisons among more than 2 groups were performed by using a Kruskal − Wallis test, followed by a Steel test. The incidence of IAs or SAH was analyzed by using a Fisher’s exact test. A *p* value lower than 0.05 was defined as statistically significant.

### Ethics approval and consent to participate

All of the following experiments, including animal care and use, complied with the National Institutes of Health Guide for the Care and Use of Laboratory Animals and the Animal Research Reporting In Vivo Experiments (ARRIVE) guidelines and were approved by the Institutional Animal Care and Use Committee of National Cerebral and Cardiovascular Center (Approval No. 17085, 18010, 19036).

## Results

### Up-representation of cascades related with neutrophils in RNA sequencing analysis

**Figure 1:**
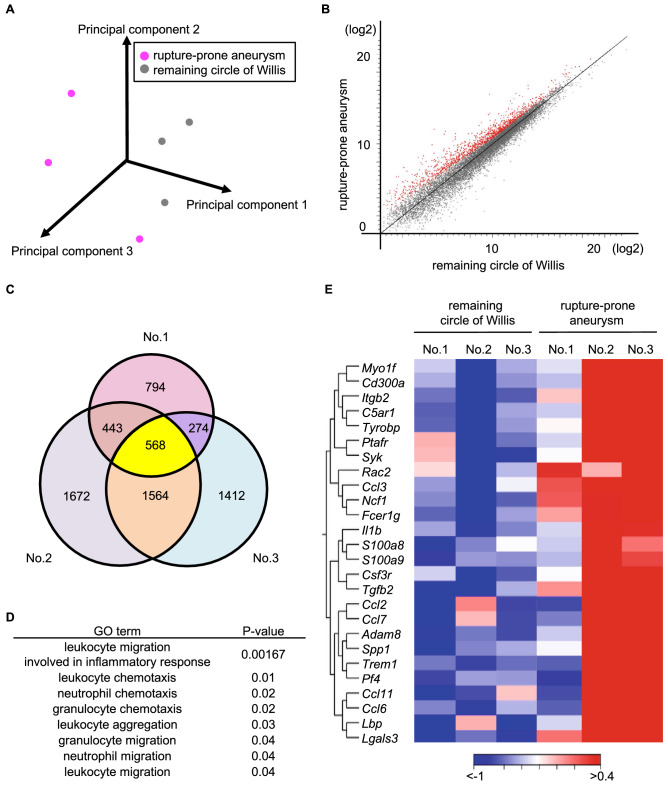
Comprehensive gene expression profile analysis of rupture-prone IAs and the remaining circle of Willis. (**A**) The principal component analysis of comprehensive gene expression profile data from rupture-prone IAs and the remaining circle of Willis (n = 3). (**B**) The scatterplot showing the over-expressed genes (shown in red) in rupture-prone IAs compared to those in the remaining circle of Willis. (**C**) The Venn diagram showing the over-expressed genes in each rupture-prone lesion compared to those in the remaining circle of Willis from a same animal (n = 3). Yellow color indicates genes over-expressed in all rupture-prone lesions in common. (**D**) Identification of neutrophil-related terms in gene ontology analysis using 568 over-expressed genes. (**E**) The heat map showing gene expression profile of over-expressed genes in each rupture prone lesion and the remaining circle of Willis.

Fifteen rats were subjected to surgical manipulations to induce IAs^4^ and, at the 63rd days after manipulations, 5 of them developed IAs at the anterior- or posterior communicating artery; among them, one was ruptured and the other 4 were unruptured lesions. From these 4 rats with unruptured IAs, the circle of Willis was stripped and unruptured lesions at the anterior or posterior communicating artery were harvested as a rupture-prone lesion (Supplementary Fig. S1). The total RNA samples that were purified from dissected IA tissues and the remaining circle of Willis were then subjected to RNA sequencing analyses to obtain gene expression profile data and to identify the cascades that were specifically up-represented in rupture-prone IA lesions, compared with those in the remaining circle of Willis. RNA samples from one IA specimen and the corresponding remaining circle of Willis were excluded from the analysis, along with the corresponding circle of Willis, because of the low RNA yield. The median RIN value of RNA samples used in RNA sequencing analyses was 7.9 and 8.1 (n = 6). A total of 161—176 million reads mapped in pairs were obtained in the present experiment. Among these, 88.1% of fragments per sample, on average, were successfully mapped to the reference rat genome (Rnor6). Principal component analysis demonstrated variability among the rupture-prone IA samples, suggesting the heterogeneity of the lesions (Fig. 1A). Considering the heterogeneity of the lesions (Fig. 1A) and the finding that more than half of rupture-prone lesions will rupture^4^, we selected the genes that were over-expressed in all the IA samples, compared with those in the remaining circle of Willis. We then identified a couple of differentially expressing genes, 568 over-expressed and 961 under-expressed ones, in rupture-prone IAs, compared with those in the remaining arterial walls in the circle of Willis (Fig. 1B and 1C, Supplementary Table S2 and S3). We used GO analysis to analyze 568 over-expressed genes by using the CLC genomics workbench system, from which 228 terms were identified as being up-represented in IA lesions. Intriguingly, these terms included several neutrophil- or leukocyte-related ones (Fig. 1D), suggesting the role of neutrophils in the process of regulating the rupture of lesions. In the heat map, individual variabilities were observed, as is the case with principal component analysis (Fig. 1A). Some genes that were over-expressed in rupture-prone IAs were associated with some chemoattractants of inflammatory cells, such as *Ccl2* and *Ccl3*, *Ccl6, Ccl7* and cytokines, such as *Il1b* (Fig. 1E). Because some of these factors play a role in the pathogenesis of IAs^8–10^, the contribution of neutrophils to the pathogenesis of IAs in terms of inflammation is further supported.

### Accumulation of neutrophils around the site of rupture in IA lesions

**Figure 2:**
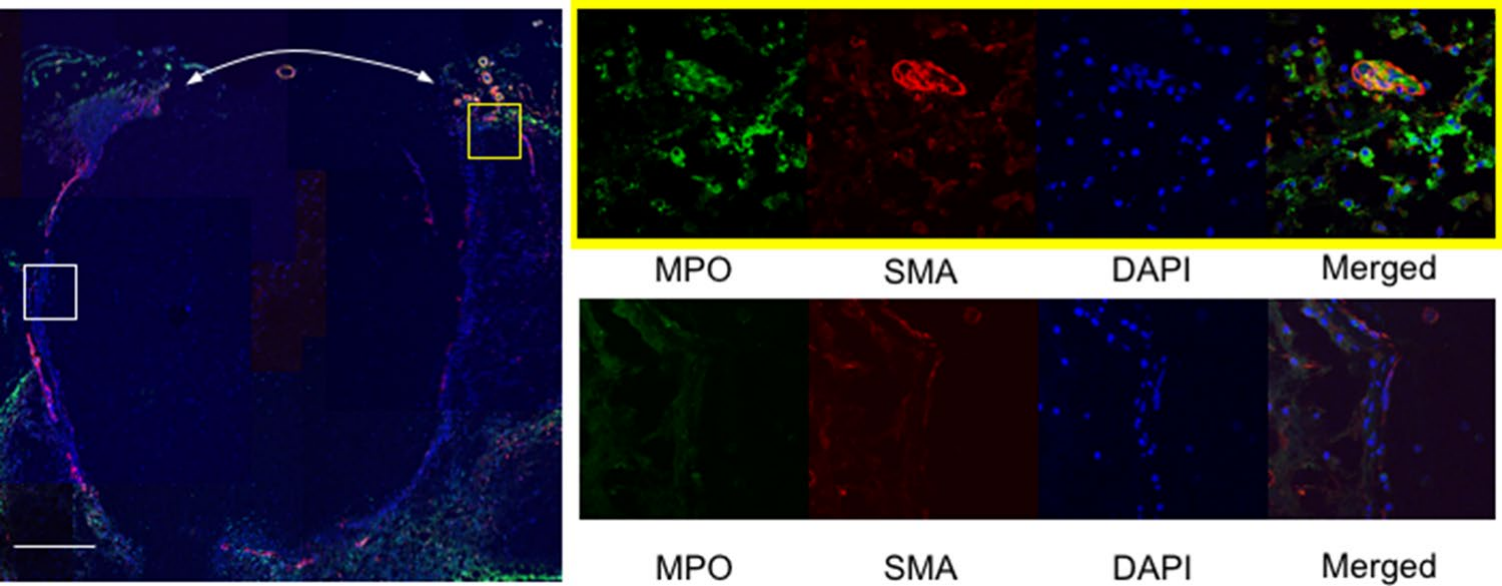
Accumulation of neutrophils around the site of rupture in IA lesions. Abundant infiltration of myeloperoxidase (MPO)-positive cells and predominance of the presence of vasa vasorum with α-smooth muscle actin (SMA) positive media around the site of rupture. The representative images of immunostaining for MPO (green), SMA (red), of nuclear staining by DAPI (blue) and merged images are shown. The magnified images corresponding to the squares in the left panel are shown on the right. The arrows indicate the site of rupture. Bar; 100 μm.

Next, we examined whether neutrophils could, indeed, be detected in IA lesions, especially ruptured ones in immunohistochemistry, as a marker of neutrophils, MPO. By using immunohistochemical examination, the accumulation of MPO-positive cells, neutrophils, was observed in the ruptured IA lesions of the rat model, almost strictly around the site of rupture, although those cells could be rarely detected in other parts of the aneurysmal walls (Fig. 2). Note that there were some vasa vasorum with the SMA-positive medial smooth muscle cell layer, specifically around the site of rupture and there was an accumulation of neutrophils nearby (Fig. 2), suggesting the infiltration of neutrophils through the vasa vasorum. Here, we have recently clarified the association of the induction of vasa vasorum formation with the rupture of IAs, presumably via facilitating the infiltration of inflammatory cells^4^. Combined together, these results indicate that the accumulation of neutrophils through vasa vasorum exacerbates the inflammatory response at the prospective site of rupture and the resultant degenerative changes of the arterial walls in situ lead to the rupture of IAs. This way, we hypothesized that the machinery mediating the rupture of lesions was driven only by a tiny area of the IA walls.

### Effect of the increase in the number of neutrophils in lesions on the rupture of IAs

**Figure 3:**
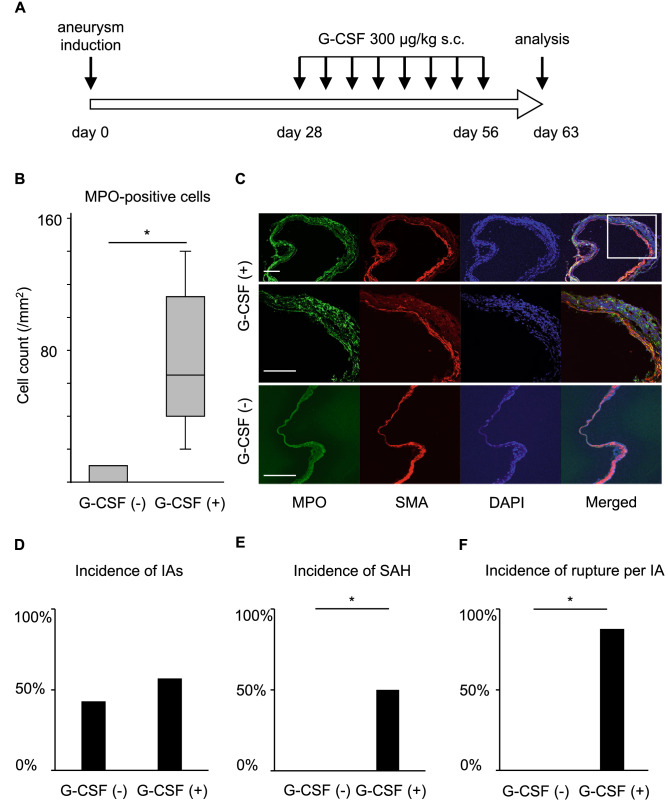
Administration of G-CSF and the increase in the number of neutrophils in IA lesions. (**A**) Time course of the experiment. (**B**,**C**) Increase of myeloperoxidase (MPO)-positive cells infiltrating in IA walls of rats treated with G-CSF. IA lesions were harvested from rats subjected to an IA model and treated with G-CSF (300 μg/kg, G-CSF ( +), n = 14) or vehicle (G-CSF ( −), n = 7) and immunostained. The cell count of MPO-positive cells, neutrophils, is shown in (**B**). Statistical analysis was done by a Mann–Whitney *U* test. *; *p* < 0.05. The representative images of immunostaining for MPO (green), α-smooth muscle actin (SMA, red), of nuclear staining by DAPI (blue) and merged images are shown in (**C**). Bar; 100 μm. (**D**–**F**) Effect of increased neutrophils by treatment with G-CSF on incidence or rupture of IAs. Rats were subjected to IA induction and treated with G-CSF as shown in (**A**). The incidence or rupture of IAs at the anterior- or posterior-communicating artery was examined (vehicle-treated group, n = 7, G-CSF-treated group, n = 14). Statistical analysis was done by a Fisher’s exact test. *; *p* < 0.05.

**Figure 4:**
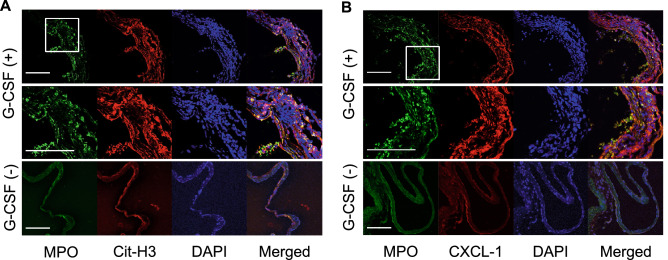
Activation and CXCL-1 induction in neutrophils in lesions from a rat model treated with G-CSF. IA lesions were harvested from rats subjected to an IA model and treated with G-CSF (300 μg/kg, G-CSF ( +)) or vehicle (G-CSF ( −)) as shown in Fig. 3A and immunostained. The representative images of immunostaining for myeloperoxidase (MPO, green), citrullinated Histone H3 (Cit-H3, red in **A**), CXCL-1 (red in **B**), of nuclear staining by DAPI (blue) and merged images are shown. The magnified images corresponding to the squares in the upper panels are shown in the lower panels. Bar; 100 μm.

To corroborate the contribution of neutrophils and neutrophil-mediated machineries on the rupture of IAs, G-CSF was administered to a rat model to increase the number of neutrophils infiltrating the lesions (Fig. 3A). The administration of G-CSF (300 μg/kg) to the model increased the number of neutrophils infiltrating the IA lesions by over 20-fold and the differences reached statistically significance (Vehicle-treated group, minimum 0/mm^2^, lower quartile: 0/mm^2^ median: 0/mm^2^, upper quartile: 10/mm^2^, maximum: 10/mm^2^, n = 3; G-CSF-treated group, minimum 20/mm^2^, lower quartile: 40/mm^2^, median: 65/mm^2^, upper quartile: 112/mm^2^, maximum: 140/mm^2^, n = 8; Vehicle-treated vs. G-CSF-treated group: *p* = 0.018) (Fig. 3B and 3C). Twenty-one rats were subjected to IA induction; 14 of them were treated with G-CSF (300 μg/kg, twice/week, s. c.) and the remaining 7 rats were allocated to the vehicle-treated group. Eight aneurysms in 14 G-CSF-treated rats (57.1%) or 3 aneurysms in 7 vehicle-treated rats (42.9%) were induced at the anterior- or posterior communicating artery at the 63rd day after the induction. G-CSF treatment did not influence the incidence of IAs (Fig. 3D). Importantly, the incidence of SAH in G-CSF-treated rats (7/14 rats, 50%; 7/8 aneurysms, 87.5%) was significantly higher than that in the vehicle-treated group (0/7 rats; 0/3 aneurysms) (Fig. 3E and 3F). In immunohistochemistry, most MPO-positive neutrophils were positive for citrullinated histone H3 (Cit-H3) (Fig. 4A), a marker of the neutrophil extracellular traps, indicating the activation and the presence of NETosis^11,12^, presumably due to cytokines present in the microenvironment. Consistently, most positive signals for MPO in immunohistochemistry were also positive for CXCL-1 (Fig. 4B), a cytokine with chemotactic activity for neutrophils^13,14^, suggesting the formation of an auto-amplification loop for an in situ accumulation of neutrophils, which was observed in the present experiment (Fig. 2), and also for macrophages in IAs^7^. Although neutrophils were accumulated and activated in IA walls, specifically around the site of rupture, the number of infiltrated macrophages, a cell type that significantly contributes to the formation and progression of the disease^8,9,15,16^, in whole lesions was not different between the G-CSF-treated and the vehicle-treated groups (Vehicle-treated group, minimum 0/mm^2^, lower quartile: 0/mm^2^, median: 110/mm^2^, upper quartile: 120/mm^2^, maximum: 120/mm^2^, n = 3; G-CSF-treated group, minimum 0/mm^2^, lower quartile: 0/mm^2^, median: 15/mm^2^, upper quartile: 167/mm^2^, maximum 330/mm^2^, n = 8) (Supplementary Fig. S2). Considering that neutrophils accumulated specifically in a tiny area of the IA lesions (Fig. 2) but could facilitate rupture (Fig. 3), neutrophils might function as facilitators of the degenerative changes of the arterial walls only in the microenvironment around the site of rupture.

### Contribution of neutrophils to the rupture of IAs

**Figure 5:**
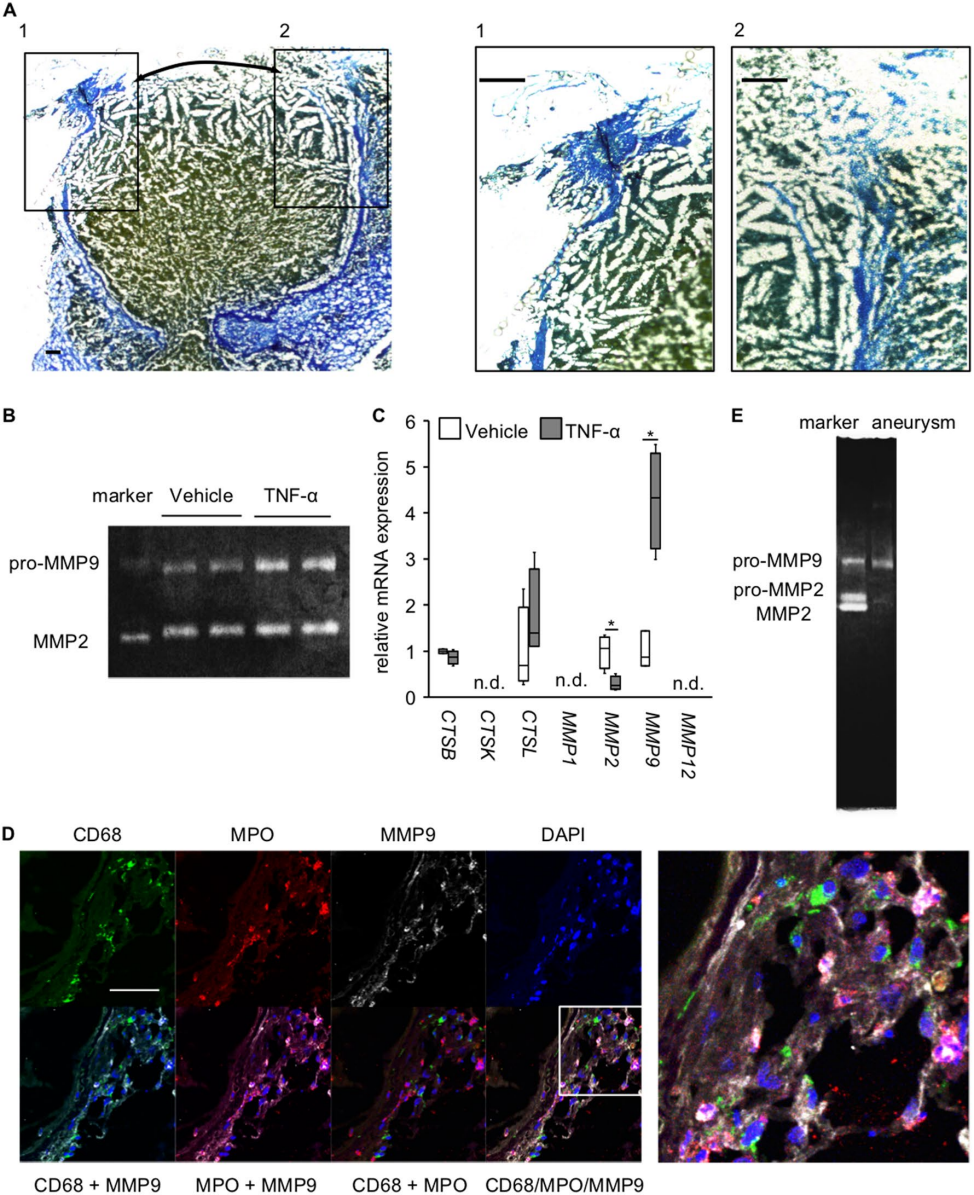
Production of MMP9 from neutrophils infiltrating in IA lesions. (**A**) The degenerative changes of collagens around the site of rupture in IA lesions. The representative images of AZAN staining of IA lesions induced in a rat model are shown. Arrows in the left panel indicate the site of rupture. The magnified images corresponding the squares in the left panel are also shown on the right. Bar; 100 μm. (**B**) Collagenolytic activity of neutrophils. Cultured neutrophils (HL-60 cells) were stimulated with recombinant TNF-α (100 μg/ml, 5 h) and the collagenolytic activity in the supernatant was examined by a gelatin zymography using recombinant pro-MMP9, pro-MMP2 and MMP2 as a reference. The representative image of the gel from a gelatin zymography from 3 independent experiments is shown. The raw image of the gel is shown in Supplementary Figure S3. (**C**) Induction of *MMP9* by TNF-α in neutrophils. Neutrophils were stimulated with recombinant TNF-α (100 ng/ml, 90 min) and the expression of proteinases was examined in RT-PCR analysis (n = 4, except vehicle of MMP9; n = 3). Statistical analysis was done by a Mann–Whitney *U* test. *; *p* < 0.05. n.d.; not detectable. (**D**) Expression of MMP9 in neutrophils infiltrating in lesions. IA lesions were harvested from rats subjected to an IA model and immunostained. The representative images of immunostaining for a macrophage marker, CD68, (green), myeloperoxidase (MPO, red), MMP9 (white), of nuclear staining by DAPI (blue) and merged images are shown. The magnified image corresponding to the square is shown on the right. Bar; 50 μm. (**E**) Detection of the collagenolytic activity of MMP9 in ruptured IA lesion. Ruptured IA lesions were harvested from rats subjected to an IA model and grinded. Collagenolytic activity was then examined by a gelatin zymography using recombinant pro-MMP9, pro-MMP2 and MMP2 as a reference. The representative image of the gel from a gelatin zymography is shown.

In AZAN staining of ruptured IA lesions, destructive changes in connective tissues were most remarkable just around the site of rupture (Fig. 5A). Thereby, we then examined whether neutrophils could directly degenerate the extracellular matrix, mainly collagens, using HL-60 cells. Gelatin zymography, that was used to detect the collagenolytic activity, revealed the presence of collagenase activity in the supernatant of cultured neutrophils and this activity was, presumably, derived from MMP2 and MMP9, and the recombinant MMP2 and MMP9 served as a control (Fig. 5B and Supplementary Fig. S3). Note that no other bands, other than the bands corresponding to MMP2 and MMP9, were visible in the gel (Supplementary Fig. S3). Importantly, the collagenase activity of MMP9 was remarkably enhanced under the inflammatory stimuli by TNF-α, which is present in IA lesions and contributes to the pathogenesis^17^. Consistently, MMP9 mRNA expression was, specifically, among the proteinases examined and was significantly induced under TNF-α stimulation (*CTSB*: Vehicle-treated group, minimum: 0.9506, lower quartile: 0.9523, median: 0.9999, upper quartile: 1.0478, maximum: 1.0495, n = 4; TNF-α-treated group, minimum: 0.6826, lower quartile: 0.7287, median: 0.8739, upper quartile: 0.9964, maximum: 1.0349, n = 4; *CTSL*: Vehicle-treated group, minimum: 0.2790, lower quartile: 0.3601, median: 0.6884, upper quartile: 1.9516, maximum: 2.3443, n = 4; TNF-α-treated group, minimum: 1.1050, lower quartile: 1.1065, median: 1.4009, upper quartile: 2.7802, maximum: 3.1433, n = 4; *MMP2*: Vehicle-treated group, minimum: 0.5153, lower quartile: 0.6279, median: 1.0665, upper quartile: 1.3055, maximum: 1.3517, n = 4; TNF-α-treated group, minimum: 0.2410, lower quartile: 0.1785, median: 0.2610, upper quartile: 0.4521, maximum: 0.5091, n = 4; *MMP9*: Vehicle-treated group, minimum: 0.6851, lower quartile: 0.6851,median: 0.8763, upper quartile: 1.4386, maximum: 1.4386, n = 3; TNF-α-treated group, minimum: 2.9901, lower quartile: 3.2275, median: 4.3312, upper quartile: 5.296, maximum: 5.4867, n = 4; Vehicle-treated vs. TNF-α-treated group, *MMP2*: *p* = 0.0209, *MMP9*: *p* = 0.0339) (Fig. 5C). We next examined whether neutrophils accumulating around the site of rupture produced MMP9 in vivo using immunohistochemistry. MMP9 expression was, indeed, detected in IA walls and most of the positive signals for MMP9 in immunostaining were co-localized with those of a marker of neutrophils, MPO (Fig. 5D). Importantly and consistently, the collagenolytic activity of MMP9 was detected in ruptured lesions (Fig. 5E), suggesting the in vivo relevance of the above in vitro study. Also in neutrophils, the expression of pro-inflammatory genes, *PTGS2*, *IL6*, and *TNF*, was induced under the stimulation with TNF-α (*PTGS2*: Vehicle-treated group, minimum: 0.897, lower quartile: 0.917, median: 1.006, upper quartile: 1.077, maximum: 1.091, n = 4; TNF-α-treated group, minimum: 1.799, lower quartile: 1.810, median: 1.855, upper quartile: 2.070, maximum: 2.138, n = 4; *IL6*: Vehicle-treated group, minimum: 0.001, lower quartile: 0.222, median: 1.201, upper quartile: 1.576, maximum:1.596, n = 4; TNF-α-treated group, minimum: 15.763, lower quartile: 15.764, median: 19.217, upper quartile: 37.471, maximum: 42.406, n = 4; *TNF*: Vehicle-treated group, minimum: 0.675, lower quartile: 0.705, median: 1.023, upper quartile: 1.272, maximum: 1.278, n = 4; TNF-α-treated group, minimum: 2.830, lower quartile: 2.847, median: 3.112, upper quartile: 4.091, maximum: 4.342, n = 4; Vehicle-treated vs. TNF-α-treated group: *PTGS2*: *p* = 0.0209, *IL6*: *p* = 0.0202, *TNF*: *p* = 0.0209) (Supplementary Fig. S4a) and the secretion of Prostaglandin E_2_ and TNF-α in the culture supernatant was consistently enhanced (PGE_2_: Vehicle-treated group without Indomethacin pre-treatment, minimum: 70.9 pg/mL, lower quartile: 76.3 pg/mL, median: 81.3 pg/mL, upper quartile: 82.0 pg/mL, maximum: 89.3 pg/mL, n = 7; Vehicle-treated group with Indomethacin pre-treatment, minimum: 48.3 pg/mL, lower quartile: 48.4 pg/mL, median: 48.8 pg/mL, upper quartile: 49. 2 pg/mL, maximum: 49.2 pg/mL, n = 4; TNF-α-treated group without Indomethacin pre-treatment, minimum: 101.6 pg/mL, lower quartile: 104.3 pg/mL, median: 107.0 pg/mL, upper quartile: 109.9 pg/mL, maximum: 124.2 pg/mL, n = 7; TNF-α-treated group with Indomethacin pre-treatment, minimum: 44.7 pg/mL, lower quartile: 44.7 pg/mL, median: 46.3 pg/mL, upper quartile: 50.4 pg/mL, maximum: 51.2 pg/mL, n = 4; TNF-α: Vehicle-treated group, not detectable, n = 10; Vehicle-treated vs. TNF-α-treated group without Indomethacin pre-treatment: *p* = 0.0017; LPS-treated group, minimum: 102.2 pg/mL, lower quartile: 107.3 pg/mL, median: 110.0 pg/mL, upper quartile: 114.9 pg/mL, maximum: 123.5 pg/mL, n = 10) (Supplementary Fig. S3b,c). These results, together with the well-established concept that IA is a chronic inflammatory disease affecting intracranial arteries^7,15,18^, suggest that neutrophils, not macrophages, actively participate in, and rather trigger, the molecular machineries leading to the rupture of the lesions.

## Discussion

In the present study, by using an animal model of IAs, the contribution of neutrophils to the process that leads to the rupture of the lesions has been suggested. Because neutrophils infiltrating these lesions produce tissue-destructive proteinases, such as MMP9 (Fig. 5D), neutrophils accumulated in microenvironment can directly exacerbate the degenerative changes of atrial walls and, presumably, facilitate the rupture of the lesions. In addition, as demonstrated in in vitro experiments (Supplementary Fig. S4), neutrophils can produce a large amount of pro-inflammatory factors, such as TNF-α and PGE_2_, in response to the cytokines present in situ, to provide an inflammatory microenvironment and to exacerbate the inflammatory response, presumably in concert with macrophages activated by neutrophil-producing cytokines. Immunohistochemical analyses also support the activation of neutrophils in lesions (Fig. 4A). Intriguingly, neutrophils in lesions produce CXCL-1 (Fig. 4B) and form an auto-amplification loop to accumulate in these lesions, leading to an inflammatory microenvironment at the prospective site of rupture, as macrophages accumulate in lesions^7,16^. Considering the crucial contribution of TNF-α or PGE_2_ to the pathogenesis of IAs revealed in animal studies^7,19–21^, in addition to macrophages, neutrophils may play a pivotal role in the maintenance and exacerbation of inflammatory responses, facilitating the degenerative changes in lesions. The involvement of inflammatory responses in these lesions in the process, leading to rupture, has also been supported by observational studies using human cases, showing that the usage of drugs with anti-inflammatory effects, such as statins and non-steroidal anti-inflammatory drugs, reduces the risk of SAH due to rupture of IAs^22,23^. Some studies on human IAs have also provided evidence indicating the association of neutrophils with the progression of IAs. For example, previous histopathological studies of human specimens have suggested the infiltration of inflammatory cells, including neutrophils, in IA lesions^24,25^. The histological study enrolling three ruptured and 20 unruptured IA specimens, harvested during operation, has demonstrated that all of the three ruptured lesions and half of the unruptured ones were positive for MPO staining in immunohistochemistry^26^. Importantly, the MPO-positive group has a significantly higher risk of rupture within 5 years (2.28%), estimated using PHASES scoring system^27^, than the MPO-negative group (0.69%)^26^, suggesting the involvement of a neutrophil-mediated pathway in the process leading to rupture. In another study, 36 saccular intracranial aneurysms from human cases, 16 unruptured and 20 ruptured lesions, were immuno-stained and positive signals for MPO were detected in all aneurysm lesions^28^. Because the extent of the MPO-positive area in immunohistochemistry was significantly correlated with the degenerative changes in the arterial walls of the lesions or infiltration of inflammatory cells, including macrophages^28^, the crucial contribution of MPO-positive neutrophils in the progression of the disease is, again, implicated. Intriguingly, the study enrolling 25 human IA cases and examining the concentration of MPO in plasma from IA lesions and femoral artery has demonstrated a significant increase in the concentration of MPO in the plasma from IA lesions, compared with that from the femoral artery, supporting the role of MPO or neutrophils in the microenvironment of the lesions, for the progression of the disease. Although a careful interpretation is necessary, especially regarding whether MPO activity indeed reflects the activity of neutrophils, the genetic deletion of MPO results in the suppression of SAH in a mice model of IAs, in which lesions are induced via systemic hypertension and elastase infusion^29^, providing experimental evidence that suggests the contribution of neutrophils to the rupture of IAs.

There are some limitations in the present study. The most serious limitation is the lack of experimental evidence demonstrating the causative relationship between the rupture of induced lesions and the activity of neutrophils. The antibody-mediated depletion of neutrophils by anti-Ly6G antibody has been reported in mice^30–33^. However, we used rats for establishing an animal model and failed to find an antibody to deplete neutrophils, partially because the antibody-mediated depletion of neutrophils paradoxically increased the number of CD68-positive macrophages in lesions. The establishment of an experimental method to effectively and specifically deplete neutrophils should be achieved to accurately examine the contribution of this cell type to the pathogenesis. Also, we used G-CSF in the present study to increase the number of neutrophils, in order to examine the contribution of this type of cells to the process underlying the rupture of lesions. Since G-CSF treatment affects neutrophil function, such as via a reduction of the chemotactic activity^34^ and an increase in CD11b expression^35^, the results from G-CSF treatment show both an increase in the neutrophil number and a functional alternation. Another major limitation is the usage of HL-60 cell line, derived from a human case of acute promyelocytic leukemia, as a neutrophil model. The response of these cells to exogenous cytokines or the production of MMPs may, thus, be different from that of neutrophils in lesions or in peripheral blood. A careful interpretation is, therefore, needed.

In the present study, through in vivo and in vitro studies, the crucial role of neutrophil-mediated machineries in the rupture of IAs has been highlighted. Intriguingly, accumulating evidence, mainly through experimental studies using animal models of IAs, has revealed the involvement of macrophages and macrophage-mediated inflammatory responses in the pathogenesis of IAs, especially in the initiation and progression of the disease^7–9,16,18,36^. Considering the results from the present study and previous findings^7–9,15^, the type of cells contributing in each process to the disease development may be different; macrophages are the main contributors in initiation and progression, and neutrophils, in rupture. In a rat model of IAs, IA lesions were induced at an anterior cerebral-olfactory artery bifurcation in almost all animals investigated, but these lesions were never ruptured. On the contrary, the incidence of lesions at an anterior communication artery or posterior communicating artery bifurcation was only 50%, but these lesions have a high potential of rupture^4^. Together, these findings show that a different machinery may be involved in each step of the disease development and the contributing type of cells is presumably and accordingly different.

In the present study, we have identified the accumulation of neutrophils around the site of rupture and propose the crucial contribution of neutrophil-mediated tissue destruction and exacerbation of inflammation to the rupture of the lesions. Thus, a non-invasive imaging of the presence or the activation of this type of cells in lesions could be an ideal diagnostic method to more exactly predict the rupture of lesions or to identify rupture-prone lesions from stable ones. Nano-particle-based visualization of neutrophils using MRI as in macrophages^37–39^, may become a novel diagnostic method. Although the excellent specificity to neutrophils is essential as a diagnostic method to accurately reflect their presence or activity, the minimum specificity needed for usage as a diagnosis predictor still remains to be established.

## Conclusions

By using a rat model of IA, we have suggested the crucial role of neutrophils in the process leading to the rupture of IAs. In this process, this type of cells forms a self-amplification loop via CXCL-1 to create an inflammatory environment and facilitate degenerative changes of the arterial walls leading to the rupture of the lesions via the production of tissue-destructive proteinases, such has MMP9. Also, neutrophils could exacerbate the in situ inflammatory responses through cytokine secretion. This study has, thus, proposed neutrophils as candidates for therapeutic or diagnostic targets. Considering the poor outcome of SAH and the resultant social losses, due to the lack of medical therapy, the present study can greatly contribute, not only to the field of vascular biology, but also to public health.

## Supplementary information

Supplementary Figures.

Supplementary Table S1.

Supplementary Table S2.

Supplementary Table S3.

## Data Availability

The datasets used and/or analyzed during the current study are available from the corresponding author upon reasonable request.

## Abbreviations

G-CSF: Granulocyte-colony stimulating factor
IAs: Intracranial aneurysms
LPS: Lipopolysaccharide
MMP9: Matrix metalloproteinase 9
MPO: Myeloperoxidase
PTGS: Prostaglandin-Endoperoxide Synthase
SAH: Subarachnoid hemorrhage
SMA: Smooth muscle α-actin
TNF: Tumor necrosis factor
