# Supplementary material for: Involvement of neutrophils in machineries underlying the rupture of intracranial aneurysms in rats
Source: Sci Rep. 2020 Nov 17;10:20004. doi: 10.1038/s41598-020-74594-9 (PMC7672058; doi:10.1038/s41598-020-74594-9)
Supplement: Supplementary file 1 — Supplementary Figures. [file 41598_2020_74594_MOESM1_ESM.pdf]

Involvement of neutrophils in machineries underlying the rupture of intracranial aneurysms in rats

Mika Kushamae, Haruka Miyata, Manabu Shirai, Kampei Shimizu, Mieko Oka,
Hirokazu Koseki, Yu Abekura, Isao Ono, Kazuhiko Nozaki, Tohru Mizutani, Tomohiro Aoki

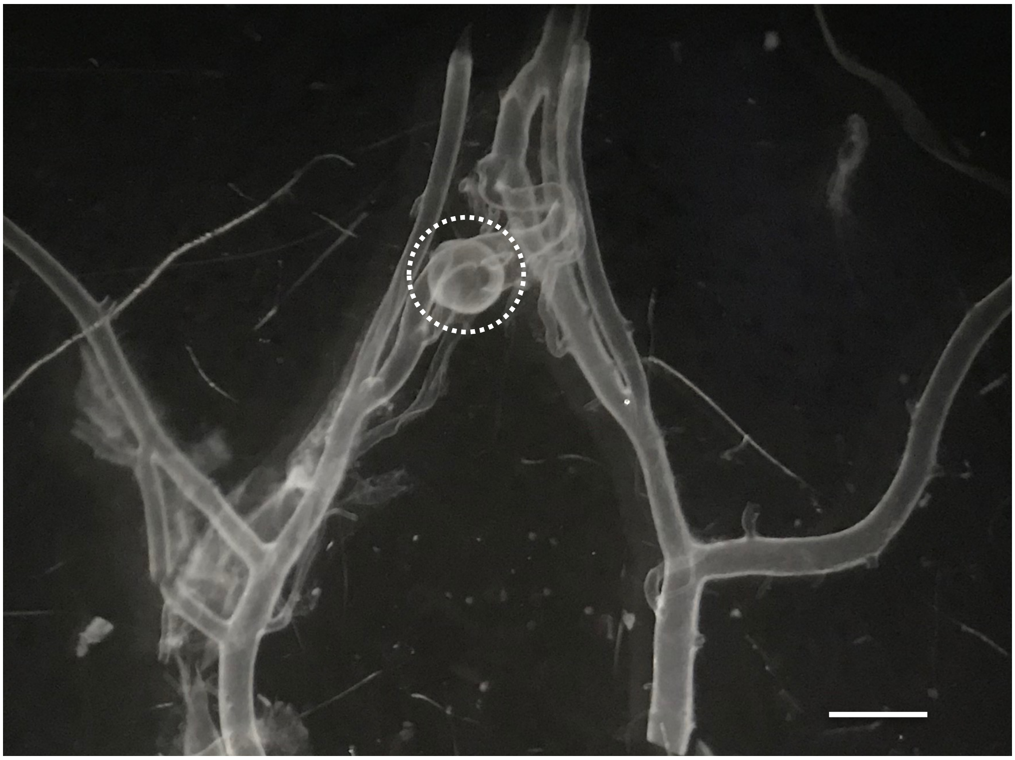

Supplementary Figure S1. IA lesion induced in a rat model
Rats were subjected to an IA model and the IA lesions including the surrounding the circle of Willis were harvested. The representative macroscopic image of the induced IA lesion (indicating by the dotted white circle) is shown. Bar; 1 mm.

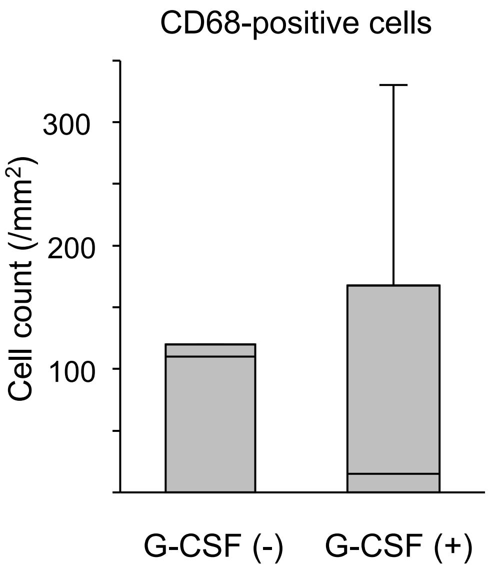

Supplementary Figure S2. Influence of G-CSF treatment on the number of CD68-positive cells infiltrating in IA lesions of rats

IA lesions were harvested from rats subjected to an IA model and treated with G-CSF (300 μg/kg, G-CSF (+), n=14) or vehicle (G-CSF (-), n=7) as shown in Fig. 3A and immunostained. The cell count of CD68-positive cells, macrophages, is shown. Statistical analysis was done by a Mann-Whitney *U* test.

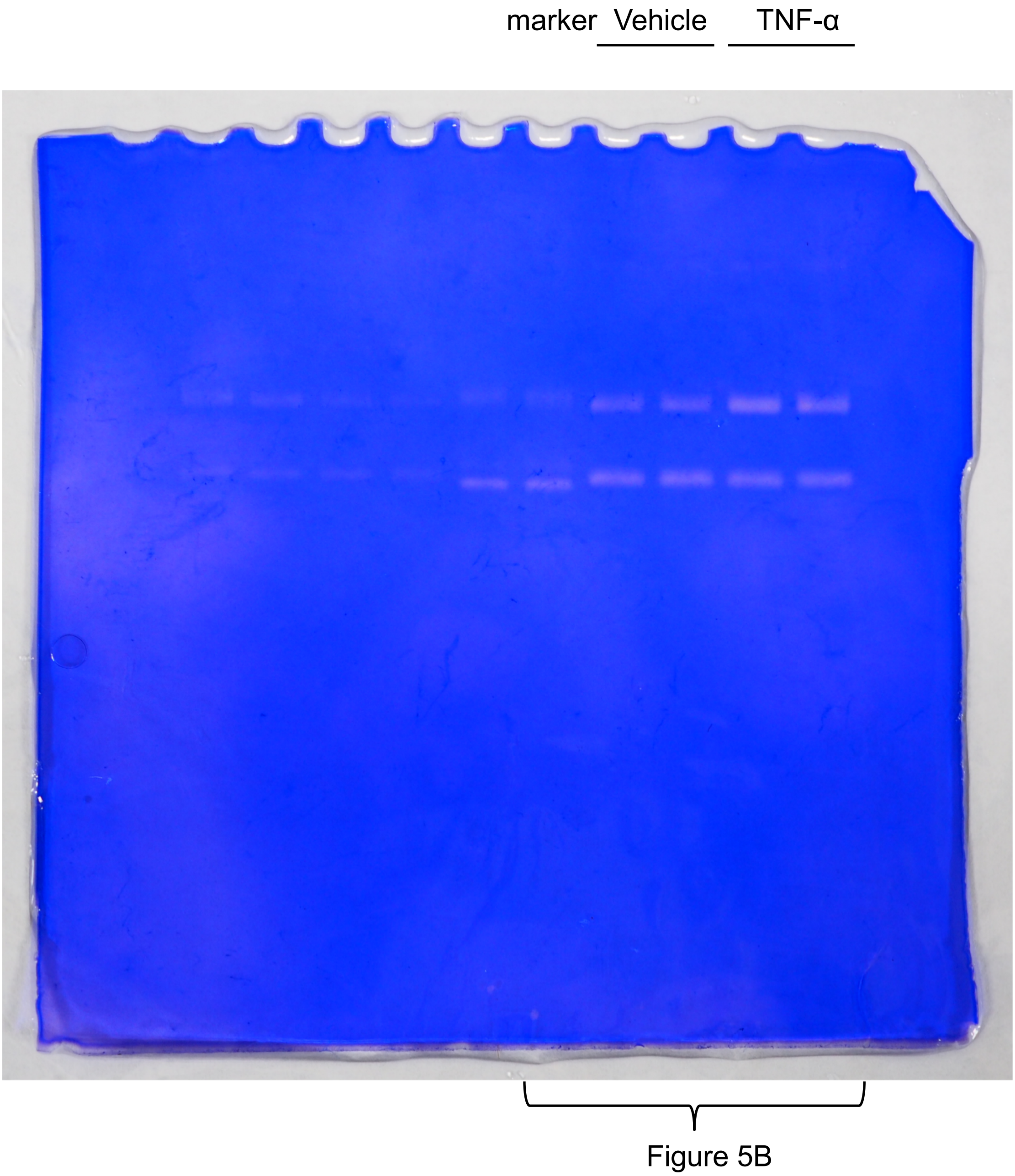

Supplementary Figure S3. The collagenolytic activity in the supernatant of cultured neutrophils examined by a gelatin zymography

Cultured neutrophils (HL-60 cells) were stimulated with recombinant TNF-α (100 ng/ml, 5 h) and the collagenolytic activity in the supernatant was examined by a gelatin zymography using recombinant pro-MMP9 and MMP2 as a reference. The raw image of the gel used in Fig. 5B is shown.

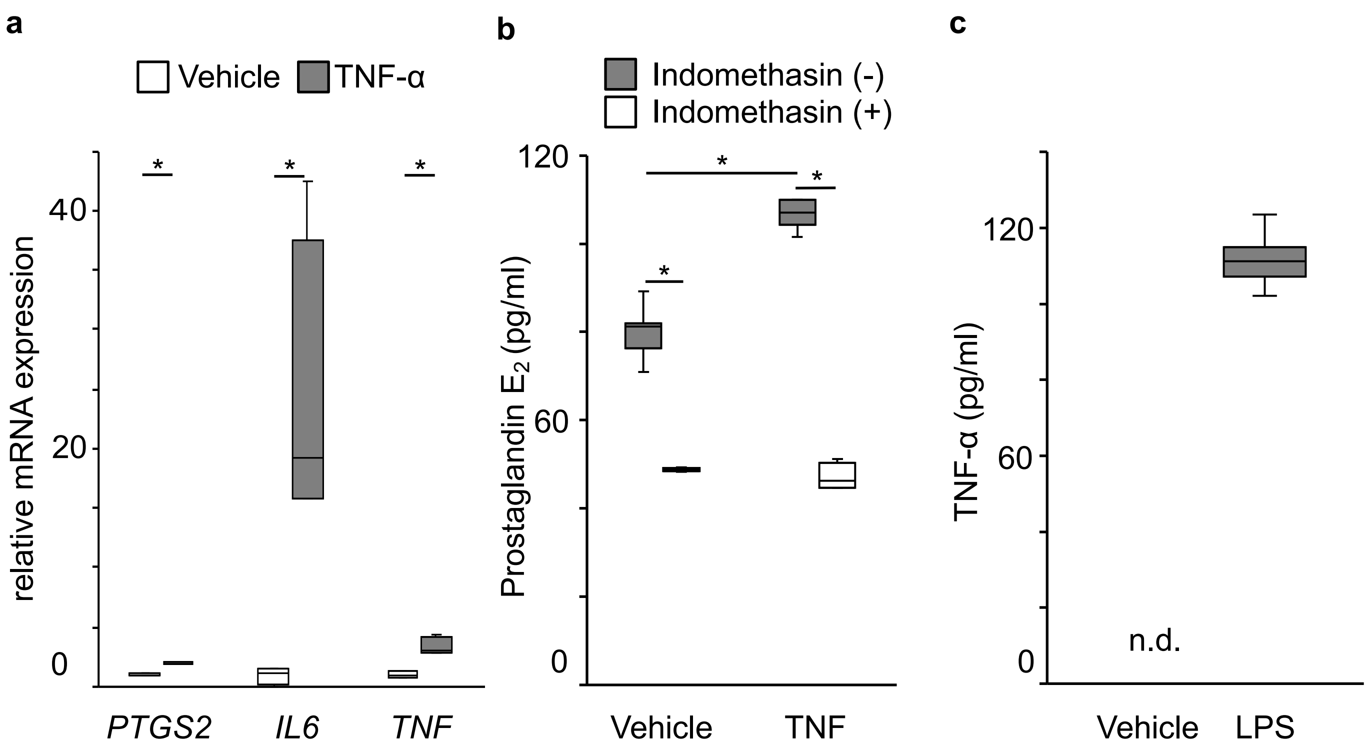

Supplementary Figure S4. Production of pro-inflammatory factors from stimulated neutrophils **a** Induction of pro-inflammatory genes by the stimulation with TNF-α in neutrophils. HL-60 cells used as neutrophils were stimulated with recombinant TNF-α (100 ng/ml, 90 min) and the expression of pro-inflammatory genes, *PTGS2* (which encodes COX-2), *IL6* and *TNF*, was examined in RT-PCR analysis (n=4). Statistical analysis was done by a Mann-Whitney *U* test. *; $p<0.05$. **b** and **c** Production of Prostaglandin E_2 or TNF-α from neutrophils under inflammatory stimuli. HL-60 cells were stimulated with recombinant TNF-α (100 ng/ml) or LPS (10 μg/ml) for 5 h and the supernatant was subjected to EIA or ELISA to measure the concentration of Prostaglandin E_2 or TNF-α, respectively. In the experiment to measure the concentration of Prostaglandin E_2, cells were pre-treated with indomethacin (100 μM) as a control study. Statistical analysis was done by a Kruskal-Wallis test (**b**, n=4;Indomethasin (+), n=7;Indomethasin (-)) or Mann-Whitney *U* test (**c**, n=10).
*; $p<0.05$. n.d.; not detectable.
